# Benzimidazole Schiff base derivatives: synthesis, characterization and antimicrobial activity

**DOI:** 10.1186/s13065-019-0642-3

**Published:** 2019-11-09

**Authors:** Thierry Youmbi Fonkui, Monisola Itohan Ikhile, Patrick Berka Njobeh, Derek Tantoh Ndinteh

**Affiliations:** 1 Department of Biotechnology and Food Technology, University of Johannesburg, Doornfontein Campus, P.O. Box 17011, Johannesburg, 2028 South Africa; 2 Department of Applied Chemistry, University of Johannesburg, Doornfontein Campus, P.O. Box 17011, Johannesburg, 2028 South Africa

**Keywords:** Schiff bases, Benzimidazole, Antibacterial, Antifungal and antiparasitic activity

## Abstract

A series of Schiff bases (**3.a**–**f**) bearing benzimidazole moiety was successfully synthesized in ethanol by refluxing Oct-2-ynoic acid (1,3-dihydrobenzimidazole-2-ylidene)amide with substituted amines. Fourier transform infrared (FTIR), ultra violet light (UV–VIS), elemental analysis, proton (^1^H) and carbon (^13^C) nuclear magnetic resonance spectroscopy were used to characterize the newly synthesized Schiff bases. Micro dilution method was used to determine the minimum inhibitory concentration (MIC) and minimum fungicidal concentration (MFC) of the Schiff bases, against 14 human pathogenic bacteria (8 Gram negative and 6 Gram positive) and against 7 fungal strains (5 *Aspergillus* and 2 *Fusarium*) representatives. Antimalarial activity against *Plasmodium falciparum* and antitrypanosomal property against *Trypanosoma brucei* was studied in vitro at a single dose concentration of the Schiff bases. Cytotoxicity of the Schiff bases was assessed against human cervix adenocarcinoma (HeLa) cells. Results obtained show that the newly synthesized Schiff bases are very potent antimicrobial agents. Gram negative bacteria *Klebsiella pneumonia* and *Escherichia coli* were more affected on exposure to Compounds **3.c**–**f** (MIC 7.8 µg/mL) which in turn exhibited more antibacterial potency than nalidixic acid reference drug that displayed MICs between 64 and 512 µg/mL against *K. pneumonia* and *E. coli* respectively. The test compounds also demonstrated high cytotoxic effect against *Aspergillus flavus* and *Aspergillus carbonarius* as they displayed MFC 7.8 and 15.6 µg/mL. Compound **3.c** exhibited the highest fungicidal property from this series with MFC alternating between 7.8 and 15.6 µg/mL against the investigated strains. The malarial activity revealed Compounds **3.c** and **3.d** as the more potent antiplasmodial compounds in this group exhibiting 95% and 85% growth inhibition respectively. The IC_50_ of Compounds **3.c** and **3.d** were determined and found to be IC_50_ 26.96 and 28.31 µg/mL respectively. Compound **3.a** was the most cytotoxic agent against HeLa cells in this group with 48% cell growth inhibition. Compounds **3.c**, **3.d** and **3.f** were biocompatible with HeLa cells and displayed low toxicity. With a very low cytotoxic effect against HeLa, compound **3.c** stands out to be a very good antiparasitic agent and consideration to further evaluate the candidate drug against others cell lines is necessary.

## Introduction

Fungi, bacteria, parasites, and viruses are at the forefront of the global health challenges as they continue to nullify the potency of many antimicrobial agents [1]. From the literature, it is clear that significant efforts to remedy the situation are available however, none can deny the current problem of microbial resistance. The prevalence of these microorganisms is a concern because of adaptations and mutations, the selectivity of the novel developing drugs and the toxicity effect of certain candidate drugs. Infections by these microorganisms affect the health status of the consumers or the host organisms that may result in loss of human life and livestock’s. This therefore encourages the continuous search for novel compounds with enhanced bioactive properties.

Schiff bases are an important class of organic compounds that show interest in industrial sectors with many biological and pharmaceutical applications. They are usually obtained by a condensation reaction between aldehydes or ketones (cyclic or linear) with primary amines (cyclic or linear) in alcoholic conditions [2]. Interest in the search for novel therapeutic Schiff bases to alleviate pathogens invasion associated with microbial resistance encourage the use of cyclic ring molecules. Aromatic-based Schiff bases have shown more potential in biological applications as a result of the free electron delocalization with the ring structure [3]. Schiff bases derived from heterocyclic rings present many advantages and detailed information are found here [4]. The most predominant heteroatoms found in organic molecules are mainly nitrogen, oxygen, and sulfur (N, O, S).

Nitrogen-containing heterocyclic compounds such as imidazole and benzimidazole form a framework of the important class of pharmacophores [5]. They have attracted the attention of many researchers as their derivatives astemizole, mebendazole, enviroxime, carbendazime have been widely used and also commercialized [6, 7]. Benzimidazole on its own alone is a benzo derivative of imidazole used in the development of therapeutic drugs. Its fused heterocyclic ring structure is crucial to the formation of nucleotides as they formed the nucleus of nitrogen bases [5]. Benzimidazole and its derivatives interact easily with biopolymers and promise to be forming good systems for the development of biologically active compounds with structural similarity to vitamin-B12 derivatives [8]. The fascinated biological activity of benzimidazole and its derivatives are seen against many human pathogens and microbial attacks. These include bacteria [9], fungi [10], and virus [11]. Schiff bases with benzimidazole moiety have also been used in DNA binding and cleavage [12, 13], they are also documented as topoisomerase inhibitors [14], with antitumor [15], and anticancer properties [16]. Their parasitic and viral properties cannot be denied as previously documented [17].

In our search for more dependable antimicrobial agents to limits microbial invasion and resistance to known drugs, we have synthesized a series of novel Schiff bases with benzimidazole scaffold and evaluated their antimicrobial activity against 14 bacterial strains, seven fungal strains and two parasites representative (*Plasmodium falciparum* and *Trypanozoma brucei*).

## Results and discussion

### Chemistry

**Scheme 1:**
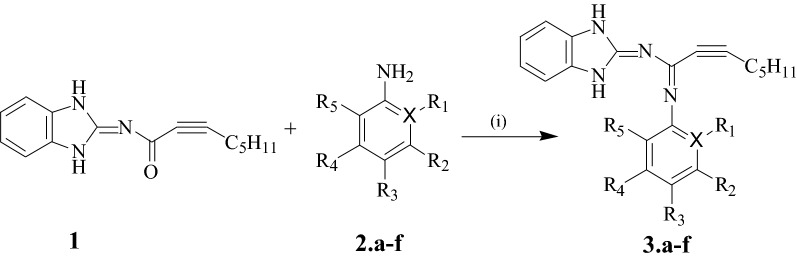
Synthesis of Schiff bases **3.a**–**f** under (i) 4 h refluxing in ethanol

Schiff base compounds **3.a**–**f** were synthesized according to the procedure shown in Scheme 1. Oct-2-ynoic acid (1,3-dihydrobenzoimidazole-2-ylidene)amide (Compound **1**) was used as the starting material. Spectral measurements including UV–VIS absorption, Fourier transform infrared (FTIR), nuclear magnetic resonance proton and carbon (^1^H and ^13^C NMR) and C, H, N elemental analysis were used to characterize and establish the structure of the prepared ligands.

Table 1 shows the physicochemical property of the Schiff bases together with their melting points and lipophilicity indexes determined on ChemDraw Ultra 7.0.

**Table 1:** Chemical property of the synthesized Schiff bases **3.a**–**f**

Carbonyl	Amines						Product	MW (g/mol)	Yield (%)	Mpt (°C)	clogP
	R_1_	R_2_	R_3_	R_4_	R_5_	X					
	NO_2_	H	H	H	H	C	**3.a**	375.42	78	178.7	5.09
	OH	H	H	H	H	C	**3.b**	346.43	46	136–137	4.68
	–	H	H	H	H	N	**3.c**	331.41	63	172.1	3.85
	H	H	COOH	H	H	C	**3.d**	374.44	73	175.2	5.09
	OH	H	SO_3_H	H	H	C	**3.e**	426.49	64	147.1	1.9
	OCF_3_	H	H	H	H	C	**3.f**	414.17	67	178–180	6.38

### Electronic spectral

**Fig. 1:**
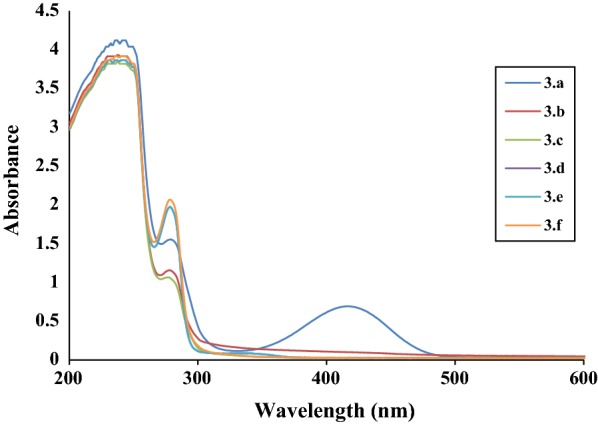
UV–vis absorption spectra of Schiff bases **3.a**–**f**

All the synthesis were carried out by mixing Compound **1** (0.3921 mmol) with aniline derivatives (0.3921 mmol) in the presence of hot ethanoic solution and reflux for 4 h to give the expected Compounds **3.a**–**f**. In order to study the spectroscopic properties of the ligands, their UV–VIS absorption spectra were recorded in DMSO and are presented in Fig. 1.

The normalized UV–VIS spectra of the compounds (Table 2) exhibit two absorption bands in low UV range (232–290 nm) except Compound **3.e** that presents an additional band at 410 nm. The bands observed below 250 nm are assigned to the π–π* transition of azomethine chromophore and above 250 nm are the typical n–π* transitions of charge transfer between C=N of the ligands [3]. The band at 410 nm observed in Compound **3.e** happened to be coming from the HSO_3_-of the aromatic ring because of the free electron distribution (Table 2).

**Table 2:** UV–vis spectroscopy of the synthesized Schiff bases **3.a**–**f**

Compounds	Absorption band (nm)	Assigned transitions
**3.a**	232–275	π–π* and n–π*
**3.b**	234–286.5	π–π* and n–π*
**3.c**	234–284	π–π* and n–π*
**3.d**	235–275	π–π* and n–π*
**3.e**	234–410	π–π* and n–π*
**3.f**	233–285	π–π* and n–π*

### Fourier transform infrared

IR spectra of this series of Schiff bases showed vibration signals at expected frequencies for the relevant functional moieties and chromophores (Table 3). The stretching frequencies at 1652–1685 cm^−1^ are due to the imine (C=N) linkage of the ligands and match similar observation here [7]. Characteristic vibration signals proper to the C–H stretching band of the aromatic ring were observed at 2955–3000 cm^−1^ with low intensity. Sharp bands present in the spectrum of the compounds, in the region 3297–1500 cm^−1^ are due to N–H stretching frequencies of imidazole moiety [18]. The vibration of C=O and S=O of Compounds **3.d** and **3.e** are seen around 2231 cm^−1^. In the spectra, no signal characteristic to the –NH_2_ of the primary amine was observed and this implies the successful formation of the Schiff base ligands.

**Table 3:** FTIR spectroscopy of the synthesized Schiff bases **3.a**–**f**

Compounds	ʋ(=C–H) (cm^−1^)	ʋ(C–N) (cm^−1^)	ʋ(C–C) (cm^−1^)	ʋ(N–H) (cm^−1^)	ʋ(N–H) (cm^−1^)	ʋ(C=N) (cm^−1^)	ʋ(C–H) (cm^−1^)
**3.a**	950	1011	1435	1438	3414	1652	3000
**3.b**	825	1030	1359	1550	3370	1677	2959
**3.c**	950	1034	1443	1501	3423	1685	2982
**3.d**	817	1034	1371	1559	3666	1684	2955
**3.e**	820	1033	1370	1541	3297	1678	2966
**3.f**	956	1035	1419	1443	3431	1685	2975

### Nuclear magnetic resonance spectroscopy

The carbonyl used in the condensation reaction was a ketone and no imine proton was observed in the spectra of the Schiff bases characterized. The ^1^H NMR of these compounds showed both aliphatic and aromatic protons (Ar–H). Aromatic protons resonate at single, doublet and multiplet at chemical shift δ = 6–7 ppm with respect to the aromatic group. However, in these compounds other Ar–H proton were seen around δ = 8 ppm (Compounds **3.a**, **3.c** and **3.f**) by reason of electron rich species on the substituted rings which in turn causes an increase in the chemical shift of the proton and result in the deshielding effect. Phenolic protons are seen at chemical shift δ = 8.2 ppm for Compound **3.b** and at δ = 10.10 ppm for Compound **3.e** as expected. Around 4 ppm chemical shift the N–H signals of the benzimidazole ring are seen. Protons of the aliphatic side chain appear as doublet, triplet and multiplet in low range chemical shift (0.8–2 ppm) with no traces of contamination. The ^13^C NMR spectra, C=N signals of the benzimidazole ring and imine of the synthesized Schiff bases were observed around δ = 158–159 ppm as expected. Aromatic carbons are seen at δ = 108–140 ppm and the alkyne carbon resonate at δ = 70–80 ppm. All aliphatic carbons were seen at δ = 13–30 ppm with C≡C–CH_2_ resonating around δ = 30 ppm.

The experimental percentage composition C, H, N of the prepared Schiff bases obtained were corresponded to the calculated data as confirmed by a difference of ± 0.6 unit seen in the recorded data.

### Pharmacological activity

#### Antibacterial activity

In vitro antibacterial activity of Schiff base compounds (**3.a**–**f**) were studied against six Gram positive and eight Gram negative bacteria using broth microdilution technique. The minimum inhibitory concentrations (MIC) of the compounds were compared to streptomycin and nalidixic acid used as reference antibiotic agents and data are presented in Table 4. Tested organisms reacted differently to ligands exposure and their susceptibility was concentration dependent. Test compounds exhibited good to high antibacterial activity against different bacterial representatives. For instance, *Staphylococcus epidermidis* was the most susceptible strain amongst the Gram positive under studied exhibiting MIC between 7.8 and 15.6 µg/mL and appears to be 8.5 times more potent than nalidixic acid (MIC 64 µg/mL). Compounds **3.a**–**c** and **3.f** displayed greater antibacterial activity (MIC 31.2 µg/mL) against *Staphylococcus aureus* than streptomycin (MIC 256 µg/mL). Gram negative bacteria *Klebsiella pneumonia* and *Escherichia coli* were more affected on exposure to Compounds **3.c**–**f** (MIC 7.8 µg/mL) which in turn exhibited more antibacterial potency than nalidixic acid that displayed MICs between 64 and 512 µg/mL against *K. pneumonia* and *E. coli* respectively. Compound **3.c** exhibited the broadest spectrum activity in this series due to the heterocyclic ring of the amine.

**Table 4:** Antibacterial activity of the Schiff bases **3.a–f**

Minimum inhibitory concentration MIC (µg/mL)														
Compounds	Gram positive						Gram negative							
	BS	EF	SE	SA	BC	MS	ECL	EM	KO	EA	PM	PA	KP	EC
**3.a**	125	250	15.6	31.2	250	250	250	125	62.5	250	250	250	15.6	7.8
**3.b**	62.5	125	15.6	31.2	125	125	250	125	125	125	125	125	62.5	31.2
**3.c**	125	125	7.8	31.2	125	125	125	125	125	125	125	125	7.8	7.8
**3.d**	125	250	7.8	250	250	250	250	250	31.2	125	250	250	7.8	7.8
**3.e**	125	125	7.8	125	125	62.5	250	125	62.5	125	125	250	7.8	7.8
**3.f**	125	125	7.8	31.2	125	31.2	125	125	62.5	125	250	125	7.8	7.8
**STM**	16	128	8	256	32	4	512	128	16	16	128	16	64	64
**NLD**	16	> 512	64	64	32	512	16	128	8	256	32	128	64	512

STM, streptomycin; NLD, nalidixic acid, BC, *Bacillus cereus*; BS, *B. subtilis*; *EF*, *Enterococcus faecalis*; MS, *Mycobacterium smegmatis*; SE, *Staphylococcus epidermidis*; SA, *S. aureus*; ECL, *Enterobacter cloacae*; EC, *Escherichia coli*; EA, *Enterobacter aerogenes*; PV, *Proteus vulgaris*; KO, *Klebsiella oxytoca*; KP, *K. pneumonia*; PM, *Proteus mirabilis*; PA, *Pseudomonas aeruginosa*

The improved potency of these compounds could also be due to other parameters besides the imine C=N bond. These include the benzimidazole ring and the substituted aromatic rings. Positive Gram bacteria were more sensitive to test compounds than negative Gram bacteria due to the nature of the cell membranes. The lack of activity may also be due to poor target engagement or non-essentiality of the targeted enzyme/partway in vivo [19]. The reason probably lies in the difference in cell membrane compositions. Unlike Gram-negative bacteria that have three other components outside the peptidoglycan (lipopolysaccharides, phospholipids, periplasmic space) for defence, positive Gram bacteria lack these protective coats outside peptidoglycan layer, which then make them more vulnerable to foreign attacks [20]. Resistance of bacteria to the synthesized Schiff bases could also be associated to the enzymatic degradation of the synthesized compounds, alteration of the bacterial protein targeted by the prepared compounds and/or change in the membrane permeability to the tested compounds [21]. In addition, Gram-negative bacteria are very much troublesome and turn to nullify the effects of almost all antibiotics and antimicrobial options available [22].

These molecules form the bilayer membrane that controls and regulate the flux of molecules in the cells; the complex lipids contain of Gram-negative bacteria may prevent the easy diffusion of chemicals into the cytoplasm of the organisms, which may not be the case of Gram-positive cells. This, therefore, makes them more resistant to chemicals compared to Gram-positive bacteria.

#### Antifungal activity

In assessing the antifungal property of the synthesized Schiff bases, five *Aspergillus* representatives and two *Fusarium* strains were considered. Minimum fungicidal concentration (MFC) by broth dilution technique was used and results are compared with amphotericin B and nystatin used as positive controls (Table 5). The compounds showed moderate to greater antifungal activity against the studied strains and to some extent exhibited antifungal greater than the standard drugs used. All test compounds showed significant antifungal activity (MFC 15.6 µg/mL) than amphotericin B (MFC 125 µg/mL) against *Aspergillus carbonarius*. This infers them preference over amphotericin B. The test compounds also demonstrated high cytotoxic effect against *Aspergillus flavus* and *A. carbonarius* as they exhibited MFC 7.8 and 15.6 µg/mL. Compound **3.c** exhibited the highest fungicidal property from this series with MFC alternating between 7.8 and 15.6 µg/mL against the investigated strains. This could be due to the heterocyclic ring and the hydrophobic nature of the compound.

**Table 5:** Minimum fungicidal concentration (MFC) of Schiff bases **3.a**–**f**

Strains code	Test compounds with MFC (µg/mL)						Standards (µg/mL)	
	**3.a**	**3.b**	**3.c**	**3.d**	**3.e**	**3.f**	AMB	NYT
ACA	31.2	15.6	15.6	15.6	15.6	15.6	125	< 8
AFL	31.2	31.2	15.6	15.6	31.2	7.8	> 8	16
AFU	7.8	7.8	7.8	7.8	7.8	15.6	16	16
ANI	15.6	31.2	15.6	15.6	31.2	31.2	16	62
APA	31.2	31.2	7.8	15.6	15.6	15.6	62	16
FPR	15.6	62.5	15.6	15.6	15.6	31.2	62	< 8
FVE	62.5	62.5	62.5	62.5	62.5	62.5	16	< 8

ACA, *Aspergillus carbonarius*; AFL, *Aspergillus flavus*; AFU, *A. fumigatus*; ANI, *A. niger*; APA, *A. parasiticus*; FPR, *F. proliferatum*; FVE, *F. verticillioides*; AMB, amphotericin B; NTY, nystatin

The interaction of synthetic chemical and natural occurring molecules with the microbial cell membrane is enhanced by their physicochemical properties. The passage or movement of chemicals and other molecules into the cytoplasm of a cell is controlled by the cell membrane via their components interacting with these molecules. The hydrophobic nature of microbial cell membrane will drive in the molecule of same nature. Lipophilicity (clogP) is an important factor that controls this phenomenon was determined. It is usually expected that a high clogP value will exhibit more antimicrobial potency but we observed in this study that the activity of these series of Schiff bases depended not on their experimental lipophilicity values but to other factors or parameters such as ligand’s geometrical arrangement, polarity, and stability. The nitro (–NO_2_) containing Compound **3.a** exhibited better antibacterial activity than its fluorinate (–OCF_3_) Compound **3.e** both at the *ortho* position. However, their hydroxyl (–OH) analogue, Compound **3.b** demonstrated a greater fungicidal activity. The carboxyl and sulfonyl based compounds (**3.d** and **3.e**) exerted good fungicidal activity against the studied fungi displaying an equipotent activity (MFC 7.8–15.6 µg/mL) against all tested strains except in *A. flavus* and *A. niger* where MFC 31.2 µg/mL was recorded (Table 5).

#### Antimalarial activity

**Fig. 2:**
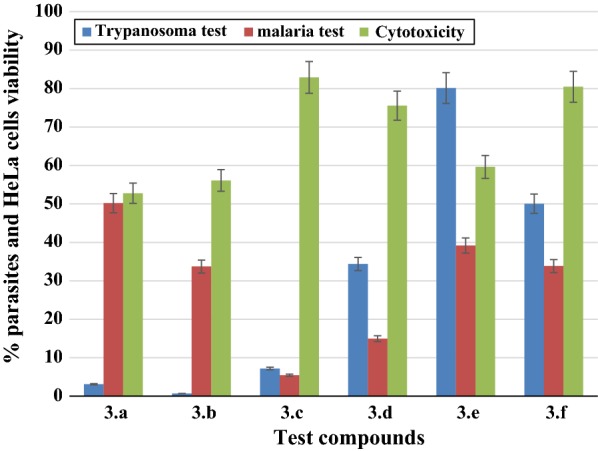
Percentage viability of *P. falciparum* strain 3D7 and *T. brucei* after 48 h exposure to the synthesized Schiff bases, together with their cytotoxicity effects against Human cervix adenocarcinoma cells (HeLa)

The antiplasmodial activity of the Compounds (**3.a**–**f**) was studied in vitro against *P. falciparum* strain 3D7. Incubation of the parasites with the test compounds for 48 h revealed their antimalarial property. All tested compounds affected the respiratory processes of the parasites and induced a significant decrease in their growth causing complete cell death at the studied concentration. These Schiff bases reduced *P. falciparum* strain 3D7 growth by more than ± 50% at a single concentration of 50 µg/mL (Fig. 2). The lowest antimalarial activity exhibited by Compound **3.a**, reduced the growth of *P. falciparum* strain 3D7 by 49%. Compounds **3.c** and **3.d** were the most potent antiplasmodial compounds in this group and exhibited 95% and 85% growth inhibition respectively. The nitro, hydroxyl, and trifluoromethoxy-substituted compounds at position two (Compounds **3.a, 3.b** and **3.f**) have good antimalarial properties with comparable antiplasmodial activity against strain 3D7. Compounds **3.b** and **3.f** have the same potency against the investigated strain though no IC_50_ was recorded. The IC_50_ (smallest concentration of compounds that reduced by 50% the growth of the parasites) of Compounds **3.c** and **3.d** were found to be IC_50_ 26.96 and 28.31 µg/mL respectively. Considering substitution at the fourth position of the aromatic ring, the carboxyl derivative Compound **3.d**, have greater antimalarial activity compared to its sulfonate analogue Compound **3.e** possibly due to the proton in *ortho* position. Compound **3.c** stand out to be most potent antiplasmodial compound investigated in this group of Schiff bases. It is important to note that the highest activity of Compound **3.c** could be due to the heterocyclic nature of the 2-aminopyrimidine. The nitrogen contain of the pyridine considerably enhanced the antimalarial activity of the compound and therefore suggests additional structure–activity relationship using the compound (**3.c**) as the parent material.

#### Antitrypanosomal test

The effects of the prepared Schiff bases on Trypanosoma parasites were assessed against *T. brucei* and data are presented in Fig. 2. All compounds were highly toxic to the studied strain except the sulfonate derivative Compound **3.e** that showed 20% loss in parasite growth in comparison to more than 50% observed with others compounds (50–92%). The nitro and hydroxyl derivatives (Compounds **3.a** and **3.b**) are highly effective against *T. brucei* compared to their trifluoromethoxy analogue Compound **3.f**. It was observed that the antitrypanosomal potency of compounds with substitution in position 2 (**3.a**, **3.b** and **3.f**) depended on the type of substituent and followed this order **3.f**, **3.a** and **3.c** with reference to their polarity. Since chemical molecules alter cell growth by causing or inducing either cytoplasmic leakage [22], disruption of respiratory processes [23, 24], binding to DNA and/or inhibit the process of replication. Cell death observed herein could also be attributed to active reactive oxygen species (ROS) induced by the tested compounds [25]. With no clear mechanism of action, the metabolic activity of the microorganism is altered by compound’s polarity (OCF_3_ > NO_2_ > OH). Structure activity relationship (SAR) in drug discovery couple different parameters; structures, geometry arrangement, and substitution (cyclic and/acyclic) and much more [26]. The hydroxyl derivative Compound **3.b** displayed the highest trypanocidal activity against *T. brucei*. The potency of this Compound **3.b** could be assigned to the –OH substitution characterized by either keto-enol tautomeric exhibited in solid state and in solution [26, 27]. Again we observed a strong trypanocidal activity exhibited by Compound **3.c** due to its pyridine contain. Pyrimidine substitution containing Schiff bases have also been recorded to exhibit antimicrobial activity against human pathogens [28–30]. This correlates well with our finding in this study.

#### Cytotoxicity test

Cytotoxicity of the compounds was evaluated against Human cervix adenocarcinoma cell (HeLa). The Schiff bases showed moderate to low toxicity against HeLa cells (Fig. 2). The percentage viability of the cells varies between 52 and 83% dependent on the compound. Compound **3.a** was the most cytotoxic agent in this group with 48% cell growth inhibition. Compounds **3.c**, **3.d** and **3.f** are biocompatible with HeLa cells as they displayed low toxicity. The heterocyclic pyridine contain Compound **3.c** has demonstrated the lowest toxic effect on HeLa cells (< 10%) and has demonstrated important antimalarial and antitrypanosomal activities. With a very low cytotoxic effect on HeLa, Compound **3.c** stands out to be a very good antiparasitic agent and consideration to further evaluate the candidate drug against others cell lines is necessary.

## Conclusion

A series of six novel Schiff base compounds with benzimidazole scaffold was successfully synthesized and their chemical structures were confirmed by UV–VIS, FTIR, elemental analysis, ^1^H and ^13^C NMR analysis. The biological applications of these compounds were studied against fourteen bacteria, seven fungi, and two parasites. The compounds have good antimicrobial activity against the studied microorganisms altering their metabolic activity and respiratory processes that lead to cell death. The newly synthesized Schiff bases have no cytotoxic effect against HeLa cell lines, and consideration for the use of these compounds as lead material for the further development of antibiotic agents is encouraged.

## Materials and methods

### Materials

Sigma Aldrich’s chemicals and reagents were purchased in South Africa and used without any additional treatment or conditioning. Benzamine derivatives such as *O*-nitroaniline, 2-aminophenol, 2-aminopyridine, *p*-aminobenzoic acid, 2-aminophenol-*p*-sulfonic acid, 2-trifluoromethoxyaniline were all purchased from the source. All microorganisms including fungi and bacteria were obtained from Davies Diagnostic South Africa. Starting material, Compound 1 (Oct-2-ynoic acid (1,3-dihydrobenzoimidazole-2-ylidene)amide) was donated by the Department of Applied Chemistry University of Johannesburg with details available online [31].

### Physical measurements

Normalized spectral data were used to ascertain, and confirm the chemical structure of the synthesized benzimidazole Schiff bases. The percentage compositions of C, H and N of the Schiff bases were determined by micro analytical method on a Flash 2000 Organic Elemental Analyzer. Fourier transform infrared (FTIR) spectra of the ligands were collected on a Spectrum 100, PerkinElmer FTIR spectrophotometer. Analysis on dried and moisture-free ligands was done on frequency range 4000–200 cm^−1^. The degradation point of the compounds was verified on an Electro thermal digital melting point apparatus that has a maximum heating capacity of 450 °C. ^1^H and ^13^C NMR spectra of the ligands were recorded in DMSO-d_6_ on a Bruker 400 MHz NMR Spectrometer operating at room temperature with tetramethylsilane (TMS) as the internal standard. Values of chemical shifts are giving in parts per million (ppm) throughout the study. A Shimadzu model UV-2540 spectrophotometer was used to record absorption spectra of the ligands suspended in DMSO at room temperature in the wavelength range of 200–800 nm.

### Synthesis of Schiff bases **3.a**–**f**

All synthesis was achieved in hot ethanol by the condensation reaction of an equimolar mixture of substituted amines (**2.a**–**f**) and Compound **1** following the procedure documented by [32]. Compound **1** (0.3921 mmol) was dissolved in ethanol and maintained warm in silicon oil. The corresponding amines (0.3921 mmol) were suspended in ethanol and added dropwise to the ethanoic solution maintained stirring and the mixture was refluxed for 4 h (Scheme 1). The colored solutions evaporated at room temperature under a fume hood and the precipitates thoroughly washed in methanol and kept dry in a desiccator.

#### *N*-*(1,3*-*Dihydro*-*benzoimidazol*-*2*-*ylidene)*-*N′*-*(2*-*nitro*-*phenyl)*-*oct*-*2*-*ynamidine (****3.a****)*

Compound **1** (100 mg: 0.3921 mmol); *O*-nitroaniline (54.16 mg: 0.3921 mmol); (yield, 78%); yellow powder, mp = 178.7 °C; UV–VIS (nm), max 232; 275. FTIR-(cm^−1^): 3414 (–OH), 3000 (C–H), 1652 (C=N), 1438 (C=C), 1011 (C–N), 950 (C–H). ^1^HNMR (400 MHz, DMSO-d_6_) δ = 8.06 (1H, s, NO_2_-Ar–H), 7.95–7.93 (1H, d, *J* = 8 Hz, NO_2_–Ar–H), 7.47–7.35 (1H, m, *J *= 8 Hz, NO_2_–Ar–H), 7.25–7.22 (2H, m, Ar–H), 7.08–7.05 (1H, m, Ar–H), 7.00–6.98 (1H, d, *J* = 8 Hz, Ar–H) 6.62–6.58 (1H, t, *J* = 8 Hz, Ar–H), 3.97 (2H, s, N–H), 2.25–2.21 (2H, t, *J* = 8 Hz, C≡CH_2_–CH-_2_), 1.45–1.28 (6H, m, CH_2_–CH_2_–CH_2_), 0.86–0.32 (3H, t, *J* = 8 Hz, CH_3_–CH_2_); ^13^CNMR (400 MHz, DMSO-d-_6_), 158.05 (C=N), 152.83 (C=N), 146.20 (C=C–N), 135.72 (NO_2_–C=C), 132.41 (C=C), 131.80 (C=C), 130.45 (C=C), 128.74 (C=C), 125.37 (C=C), 121.67 (C=C), 119.19 (C=C), 115.5 (C=C), 111.74 (C=C), 111.21 (C=C), 81.61 (C≡C), 78.56 (C≡C),30.44 (C≡C–CH_2_), 27.34 (CH_2_–CH_2_), 21.61 (CH_2_–CH_2_), 17.68 (CH_2_–CH_2_) 13,83 (CH_2_–CH_3_); Anal calcd for C-_21_H_21_N_5_O_2_ %: C, 67.18; H, 5.64; N, 18.65; O, 8.52; Found: C, 67.57; H, 6.08; N, 18.32; O, 8.09.

#### *N*-*(1,3*-*Dihydro*-*benzoimidazol*-*2*-*ylidene)*-*N′*-*(2*-*hydroxy*-*phenyl)*-*oct*-*2*-*ynamidine (****3.b****)*

Compound **1** (100 mg: 0.3921 mmol); 2-aminophenol (47.7 mg: 0.3921 mmol); (yield, 46%); brown powder, mp = 136.7–137.8 °C; UV–VIS (nm), max 234; 286. FTIR-(cm^−1^): 3370 (–OH), 2959 (C–H), 1677 (C=N), 1366 (C=C), 1030 (C–N), 825 (C–H). ^1^HNMR (400 MHz, DMSO-d_6_) δ = 8.29 (1H, s, Ar–OH), 7.95 (1H, s, Ar–H), 7.39–7.35 (2H, m, Ar–H), 6.63–6.61 (2H, m, Ar–H), 6.57–6.35 (2H, m, Ar–H), 4.61 (2H, s, N–H), 2.23–2.21 (2H, t, *J* = 8 Hz, C≡CH_2_–CH-_2_), 1.45–1.29 (6H, m, CH_2_–CH_2_–CH_2_), 0.87–0.33 (3H, t, *J* = 8 Hz, CH_3_–CH_2_); ^13^CNMR (400 MHz, DMSO-d-_6_), 157.89 (C=N), 152.99 (C=N), 144.00 (C=C–N), 136.49 (OH–C=C), 132.79 (C=C), 131.86 (C=C), 130.85 (C=C), 129.74 (C=C), 121.48 (C=C), 119.47 (C=C), 116.44 (C=C), 114.44 (C=C), 114.38 (C=C), 81.91 (C≡C), 78.26 (C≡C), 30.43 (C≡C–CH_2_), 27.36 (CH_2_–CH_2_), 21.61 (CH_2_–CH_2_), 17.68 (CH_2_–CH_2_), 13.83 (CH_2_–CH_3_); Anal calcd for C-_21_H_21_N_4_O %: C, 72.81; H, 6.40; N, 16.17; O, 4.62; Found: C, 72.08; H, 6.88; N, 16.67; O, 8.37.

#### *N*-*(1,3*-*Dihydro*-*benzoimidazol*-*2*-*ylidene)*-*N′*-*pyridin*-*2*-*yl*-*oct*-*2*-*ynamidine (****3.c****)*

Compound **1** (100 mg: 0.3921 mmol); 2-aminopyridine (36.9 mg: 0.3921 mmol); (yield, 63%); brown powder, mp = 172.1 °C; UV–VIS (nm), max 234; 284. FTIR-(cm^−1^): 3423 (–OH), 2982 (C–H), 1685 (C=N), 1438 (C=C), 1034 (C–N), 950 (C–H). ^1^HNMR (400 MHz, DMSO-d_6_) δ = 8.35 (1H, s, Ar–N–CH), 8.07 (1H, s, Ar–H), 7.86 (1H, s, Ar–H), 7.44–7.33 (1H, m, Ar–H), 7.24–7.20 (1H, m, Ar–H), 7.06–7.03 (1H, s, Ar–H), 6.45–6.41 (1H, d, *J *= 8 Hz, Ar–H), 4.83 (2H, s, N–H), 2.22–2.18 (2H, t, *J *= 8 Hz, C≡CH_2_–CH-_2_), 1.89–1.87 (2H, d, *J* = 8 Hz, CH_2_–CH_2_–CH_2_), 1.42–1.40 (2H, d, *J* = 8 Hz, CH_2_–CH_2_–CH_2_), 1.28–1.26 (2H, d, *J *= 8 Hz, CH_2_–CH_2_) 0.84–0.80 (3H, t, *J* = 8 Hz, CH_3_–CH_2_); ^13^CNMR (400 MHz, DMSO-d-_6_), 172.18 (N=C–N), 159.42 (C=N), 158.29 (C=N), 146.89 (C=C–N), 137.37 (C=C=C), 132.38 (C=C), 131.79 (C=C), 130.43 (C=C), 129.02 (C=C), 128.73 (C≡C), 121.66 (C=C), 111. 97 (C=C), 111.20 (C=C), 108.66 (C=C), 81.07 (C≡C), 78.93 (C≡C),30.44 (C≡C–CH_2_), 27.41 (CH_2_–CH_2_), 21.16 (CH_2_–CH_2_), 17.68 (CH_2_–CH_2_) 13,83 (CH_2_–CH_3_); Anal calcd for C-_20_H_21_N_5_ %: C, 72.48; H, 6.39; N, 21.13; Found: C, 72.57; H, 6.34; N, 21.09.

#### *4*-*[1*-*(1,3*-*Dihydro*-*benzoimidazol*-*2*-*ylideneamino)*-*oct*-*2*-*ynylideneamino]*-*benzoic acid (****3.d****)*

Compound **1** (100 mg: 0.3921 mmol); *p*-aminobenzoic acid (53.7 mg: 0.3921 mmol); (yield, 73%); white powder, mp = 175.2 °C; UV–VIS (nm), max 235; 275. FTIR-(cm^−1^): 3666 (–OH), 2955 (C–H), 1684 (C=N), 1481 (C=C), 1034 (C–N), 817 (C–H). ^1^HNMR (400 MHz, DMSO-d_6_) δ = 8.35 (1H, s, COOH), 7.68–7.67 (1H, d, *J* = 8 Hz Ar–H), 7.66–7.24 (1H, m, Ar–H), 7.20–7.17 (2H, m, Ar–H), 7.09–7.07 (2H, m, Ar–H), 6.71–6.69 (2H, m, Ar–H), 6.49–6.46 (2H, m, Ar–H), 4.26 (2H, s, N–H), 2.24–2.21 (2H, t, *J* = 8 Hz, C≡CH_2_–CH-_2_), 1.44–1.41 (2H, d, *J* = 8 Hz, CH_2_–CH_2_–CH_2_), 1.31–1.28 (2H, d, *J* = 8 Hz, CH_2_–CH_2_), 1.27–1.23 (2H, d, *J* = 8 Hz, CH_2_–CH_2_–CH_2_), 0.85–0.82 (3H, t, *J* = 8 Hz, CH_3_–CH_2_); ^13^CNMR (400 MHz, DMSO-d-_6_), 169.8 (C=O), 157.65 (C=N), 152.82 (C=N), 151.41 (C=C–N), 133.57 (C=C=C), 132.32 (C=C), 131.22 (C=C), 121.75 (C=C), 116.34 (C=C), 114.63 (C=C), 111.25 (C=C), 110.22 (C=C), 82.11 (C≡C), 78.33 (C≡C),30.44 (C≡C–CH_2_), 27.34 (CH_2_–CH_2_), 21.60 (CH_2_–CH_2_), 17.70 (CH_2_–CH_2_) 13,80 (CH_2_–CH_3_); Anal calcd for C_22_H_22_N_4_O_2_%: C, 70.57; H, 5.92; N, 14.96, O, 8.55; Found: C, 71.04; H, 6.16; N, 14.12; O, 8.68.

#### *4*-*[1*-*(1,3*-*Dihydro*-*benzoimidazol*-*2*-*ylideneamino)*-*oct*-*2*-*ynylideneamino]*-*3*-*hydroxy*-*benzenesulfonic acid (****3.e****)*

Compound **1** (100 mg: 0.3921 mmol); 2-aminophenol-*p*-sulfonic acid (74.1 mg: 0.3921 mmol); (yield, 64%); purplish powder, mp = 147.1 °C; UV–VIS (nm), max 234; 284. FTIR-(cm^−1^): 3297 (–OH), 2966 (C–H), 1678 (C=N), 1367 (C=C), 1033 (C–N), 820 (C–H). ^1^HNMR (400 MHz, DMSO-d_6_) δ = 10.10 (1H, s, HSO_3_), 8.40 (1H, s, OH–Ar–H), 8.23 (1H, s, Ar–H), 7.35–7.30 (1H, m, Ar–H), 7.25 (1H, s, Ar–H), 7.21–7.19 (1H, m, Ar–H), 7.10–7.08 (1H, d, *J* = 8 Hz, Ar–H), 6.74–6.72 (1H, d, *J* = 8 Hz, Ar–H), 5.87 (2H, s, N–H), 2.33–2.24 (2H, t, *J* = 8 Hz, C≡CH_2_–CH-_2_), 1.89–1.87 (2H, d, *J* = 8 Hz, CH_2_–CH_2_–CH_2_), 1.47–1.45 (2H, d, *J *= 8 Hz, CH_2_–CH_2_–CH_2_), 1.30–1.28 (2H, t, *J* = 8 Hz, CH_2_–CH_2_), 0.86–0.83 (3H, t, *J* = 8 Hz, CH_3_–CH_2_); ^13^CNMR (400 MHz, DMSO-d-_6_), 157.56 (C=N), 152.86 (C=N), 135.44 (C=C–N), 132.64 (C=C=C), 131.79 (C=C), 130.46 (C=C), 129.16 (C=C), 128.74 (C=C), 121.89 (C≡C), 121.62 (C=C), 114.34 (C=C), 112.92 (C=C), 112.43 (C=C), 111.80 (C=C), 111.27 (C=C), 82.15 (C≡C), 78.29 (C≡C), 30.43 (C≡C–CH_2_), 27.32 (CH_2_–CH_2_), 21.59 (CH_2_–CH_2_), 17.60 (CH_2_–CH_2_) 13,80 (CH_2_–CH_3_); Anal calcd for C-_21_H_22_N_4_O_4_S %: C, 59.14; H, 5.20; N, 13.14; O, 15.01; S, 7.52. Found: C, 59.62; H, 4.98; N, 13.21; O, 14.68; S, 7.51.

#### *N*-*(1,3*-*Dihydro*-*benzoimidazol*-*2*-*ylidene)*-*N′*-*(2*-*trifluoromethoxy*-*phenyl)*-*oct*-*2*-*ynamidine (****3.f****)*

Compound **1** (100 mg: 0.3921 mmol); 2-aminopyridine (36.9 mg: 0.3921 mmol); (yield, 63%); brown powder, mp = 172.1 °C; UV–VIS (nm), max 234; 284. ^1^HNMR (400 MHz, DMSO-d_6_) δ = 8.07 (1H, s, Ar–H), 7.21 (1H, m, Ar–H), 7.06–7.04 (1H, m, Ar–H), 6.45–6.41 (1H, d, *J* = 8 Hz, Ar–H), 4.50 (2H, s, N–H), 2.24–2.21 (2H, t, *J* = 8 Hz, C≡CH_2_–CH-_2_), 1.44–1.39 (2H, m, CH_2_–CH_2_–CH_2_), 1.33–1.31 (2H, d, *J *= 8 Hz, CH_2_–CH_2_–CH_2_), 1.29–1.24 (2H, m, CH_2_–CH_2_) 0.86–0.83 (3H, t, *J* = 8 Hz, CH_3_–CH_2_); ^13^CNMR (400 MHz, DMSO-d-_6_), 172.18 (N=C–N), 159.42 (C=N), 158.29 (C=N), 146.89 (C=C–N), 137.37 (C=C=C), 132.38 (C=C), 131.79 (C=C), 130.43 (C=C), 129.02 (C=C), 128.73 (C≡C), 121.66 (C=C), 111. 97 (C=C), 111.20 (C=C), 108.66 (C=C), 81.07 (C≡C), 78.93 (C≡C),30.44 (C≡C–CH_2_), 27.41 (CH_2_–CH_2_), 21.16 (CH_2_–CH_2_), 17.68 (CH_2_–CH_2_) 13,83 (CH_2_–CH_3_); Anal calcd for C_22_H_21_F_3_N_4_O %: C, 63.76; H, 5.11; F, 13.75; N, 13.52; O, 3.86 Found: C, 64.17; H, 5.78; N, 13.12; F, 13.75; O, 3.18.

### Biological activity

#### Antibacterial test

The synthesized ligands were tested in vitro against Gram negative bacterial species *Enterobacter cloacae* (**ECL**) (ATCC13047)*, E. coli* (**EC**) (ATCC25922)*, Enterobacter aerogenes*, (**EA**) (ATCC13048), *Proteus vulgaris* (**PV**) (ATCC6380), *Klebsiella oxytoca* (**KO**) (ATCC8724), *K. pneumonia* (**KP**) (ATCC13882)*, Proteus mirabilis* (**PM**) (ATCC7002) *and Pseudomonas aeruginosa* (**PA**) (ATCC27853) and Gram positive bacterial representatives *Bacillus cereus* (**BC**) (ATCC10876), *B*. *subtilis* (**BS**) (ATCC19659)*, Enterococcus faecalis* (**EF**) (ATCC13047), *Mycobacterium smegmatis* (**MS**) (MC2155), *Staphylococcus epidermidis* (**SE**) (ATCC14990) and *S. aureus* (**SA**) (ATCC25923) by microdilution method. The minimum inhibitory contractions (MIC) were determined according to reference method M38-A2 [33]. For comparison purposes, streptomycin and nalidixic acid were used as positive controls. Fresh bacterial cultures were harvested from an overnight bacterial growth and diluted in nutrient broth to match the 0.5 McFarland standards which are equivalent to 1 × 10^5^ cfu/mL. This suspension (100 µL) was then seeded under aseptic conditions in a 96-well plate containing 100 µL of serially diluted test compounds concentrations of 500 250, 125, 62.5 31.2, 15.6 and 7.8 µg/mL. The plates were then incubated at 30 °C for 24 h. Life or death cells were verified calorimetrical by addition of resazurin dye (0.02%) which is metabolized enzymatically to produce a pink resoforin precipitate in the presence of viable cells and remains blue in death cells. MIC of each ligand are recorded and presented together with the standards.

#### Antifungal test

Antifungal activity of the ligands was done the same way as mentioned above. To assess the effect of the synthesized Schiff base compounds against fungi (*A. carbonarius*, *A*. *flavus*, *A. fumigatus*, *A. parasiticus*, *A. niger*, *Fusarium proliferatum* and *F. verticillioides*), different ligand’s concentrations (500, 250, 125, 62.5 31.2, 15.6 and 7.8 µg/mL) were prepared and incubated with fresh fungal spores (1 × 10^5^ spores/mL) in a 96-well plates following the reference method for broth dilution antifungal susceptibility testing of filamentous fungi by [33]. Amphotericin B and nystatin were used throughout this study as standards fungicidal drugs. Following 72 h incubation, the plates were taking out and flooded with 10 µL (0.02%) of resazurin and the small concentrations that induced fungal cell death were considered as minimum fungicidal concentration (MFC).

#### Antiplasmodial assay

Malaria is a infectious disease caused by five different *Plasmodium* species (*P. falciparum, P. vivax, P. malariae, P. ovale,* and *P. knowlesi*) that are responsible for over 65 5000 deaths in children below the age of five years and pregnant women [34]. To evaluate the antimalarial property of the ligands synthesized, the most fatal transmitting vector (*P. falciparum* strain 3D7) was selected. The antiplasmodial activity of the ligands was done by measuring the activity of the parasite’s lactate dehydrogenase enzyme, comparing it with Chloroquine (IC-_50_ 0.01–0.05 µM) used as a positive control. Plasmodia parasites were cultured at 37 °C under an atmosphere of 5% CO_2_, 5% O_2_, 90% N_2_ in sealed T25 or T75 culture flasks for 48 h in RPMI 1640 media (2 mM l-glutamine and 25 mM Hepes (Lonza) supplemented with 5% Albumax II, 20 mM glucose, 0.65 mM hypoxanthine, 60 µg/mL gentamycin and 2–4% hematocrit human red blood cells. Two days later, the test compounds were dissolved in DMSO to the working concentration of 20 µg/L and 140 µL was transferred in 96-well plates with was then seeded with the fresh cultures prepared containing 2% parasites and incubated for another 48 h. Following this incubation period, 20 µL of this mixture was added to fresh 96-well plates conditioned with 125 µL of Malsat and NBT/PES that measures microscopically (λ_620_) the amount of purple products formed in the presence of the parasite lactate dehydrogenase (pLDH).

#### Antitrypanosomal assay

Trypanosomiasis or African sleeping sickness is an infectious disease caused by *Trypanosoma brucei* that when under looked also increases the rate of mortality in tropical hemisphere [35]. In assessing the trypanocidal property of the synthesized compounds, the ligands were suspended in DMSO to have 50 µg/mL of product and 140 µL of this solution was transferred into 96-well plate seeded with 2% cultures of *T. brucei*. The mixture was then incubated for 48 h at 37 °C under an atmosphere of 5% CO_2_, 5% O_2_, 90% N_2_. After 48 h incubation, resazurin based reagent was used to obtain the total number of parasites surviving ligand’s exposure and the results are compared with Pentamidine standard drug used as positive control.

#### Cytotoxicity assay

The toxicity of the ligands was also verified against Human cervix adenocarcinoma cell (HeLa). This was achieved by mixing equal volume of test compounds (50 µg/mL) with Hela cells (6.7 × 10^4^ cell/mL) and allowed the mixture to stand at 37 °C under an atmosphere of 5% CO_2_, 5% O_2_, 90% N_2_ for a maximum of 48 h. The numbers of Hela cells that survive compounds exposure were also determined by using resazurin based reagent and reading resorufin fluorescence in a multi-well plate reader.

## Supplementary information

**Additional file 1: Fig. S1.** FTIR spectrum of Schiff base Compound **3.a**. **Fig. S2.** FTIR spectrum of Schiff base Compound **3.b**. **Fig. S3.** FTIR spectrum of Schiff base Compound **3.c**. **Fig. S4.** FTIR spectrum of Schiff base Compound **3.d**. **Fig. S5.** FTIR spectrum of Schiff base Compound **3.e**. **Fig. S6.** FTIR spectrum of Schiff base Compound **3.f**. **Fig. S7.** Normalized UV-vis absorption spectrum of Schiff base **3.a**. **Fig. S8.** Normalized UV-vis absorption spectrum of Schiff base **3.b**. **Fig. S9.** Normalized UV-vis absorption spectrum of Schiff base **3.c**. **Fig. S10.** Normalized UV-vis absorption spectrum of Schiff base **3.d**. **Fig. S11.** Normalized UV-vis absorption spectrum of Schiff base **3.e**. **Fig. S12.** Normalized UV-vis absorption spectrum of Schiff base **3.f**. **Fig. S13.** ^1^H NMR spectrum of Schiff base Compound **3.a**. **Fig. S14.** ^13^C NMR spectrum of Schiff base Compound **3.a**. **Fig. S15.** ^1^H NMR spectrum of Schiff base Compound **3.b**. **Fig. S16.** ^13^C NMR spectrum of Schiff base Compound **3.b**. **Fig. S17.** ^1^H NMR spectrum of Schiff base Compound **3.c**. **Fig. S18.** ^13^C NMR spectrum of Schiff base Compound **3.c**. **Fig. S19.** ^1^H NMR spectrum of Schiff base Compound **3.d**. **Fig. S20.** ^13^C NMR spectrum of Schiff base Compound **3.d**. **Fig. S21.** ^1^H NMR spectrum of Schiff base Compound **3.e**. **Fig. S22.** ^13^C NMR spectrum of Schiff base compound **3.e**. **Fig. S23.** ^1^H NMR spectrum of Schiff base Compound **3.f**. **Fig. S24.** ^13^C NMR spectrum of Schiff base Compound **3.f**.

## Data Availability

All data generated or analysed during this study are included in this published article [and its Additional file 1].

## Abbreviations

ACA: *Aspergillus carbonarius*
AFL: *Aspergillus flavus*
AFU: *Aspergillus fumigatus*
AMB: amphotericin B
ANI: *Aspergillus niger*
APA: *Aspergillus parasiticus*
Ar-H: aromatic protons
*BC*: *Bacillus cereus*
*BS*: *Bacillus subtilis*
C: carbon
cfu/mL: colony forming unit per milliliter
clogP: lipophilicity
cm^−1^: wavenumber
^13^C NMR: carbon nuclear magnetic resonance spectroscopy
CO_2_: carbon dioxide
*d*: doublet
DMSO: dimethyl sulfoxide
DMSO-d_6_: deutoriated dimethyl sulfoxide
DNA: deoxyribose nucleoside triphosphate
*EA*: *Enterobacter aerogenes*
*EC*: *Escherichia coli*
*ECL*: *Enterobacter cloacae*
*EF*: *Enterococcus faecalis*
FPR: *Fusarium proliferatum*
FVE: *Fusarium verticillioides*
FTIR: Fourier Transform infrared
g/mol: gram per mole
IC_50_: smallest concentration of compounds that reduced by 50% the growth of the parasites
H: hydrogen
h: hour
^1^H NMR: proton nuclear magnetic resonance spectroscopy
HeLa: human cervix adenocarcinoma cells
Hz: hertz
*j*: the coupling constant, **J** is a measure of the interaction between a pair of protons
*KO*: *Klebsiella oxytoca*
*KP*: *Klebsiella pneumonia*
m: multiplet
MFC: minimum fungicidal concentration
mg: milli gram
MIC: minimum inhibitory concentration
MHz: mega hertz
mmol: milli mole
Mp: melting point
*MS*: *Mycobacterium smegmatis*
MW: molecular weight
N: nitrogen
nm: nanometer
*NLD*: nalidixic acid
NMR: nuclear magnetic resonance spectroscopy
NTY: nystatin
O: oxygen (atom)
O_2_: oxygen (gas)
*PA*: *Pseudomonas aeruginosa*
pLDH: parasite lactate dehydrogenase enzyme
*PM*: *Proteus mirabilis*
Ppm: parts per million
*PV*: *Proteus vulgaris*
RPMI: Roswell Park Memorial Institute
ROS: reactive oxygen species
S: sulfur
*s*: *Singlet*
*SA*: *Staphylococcus aureus*
SAR: structure activity relationship
*SE*: *Staphylococcus epidermidis*
*STM*: *Streptomycin*
t: triplet
TMS: tetramethylsilane
UV–VIS: ultraviolet visible light
µg/mL: microgram per milli liter
µL: microliter
µM: micromole
