# Supplementary material for: Benzimidazole Schiff base derivatives: synthesis, characterization and antimicrobial activity
Source: BMC Chem. 2019 Nov 9;13(1):127. doi: 10.1186/s13065-019-0642-3 (PMC6842205; doi:10.1186/s13065-019-0642-3)
Supplement: Supplementary file 1 — Additional file 1: Fig. S1. FTIR spectrum of Schiff base Compound 3.a. Fig. S2. FTIR spectrum of Schiff base Compound 3.b. Fig. S3. FTIR spectrum of Schiff base Compound 3.c. Fig. S4. FTIR spectrum of Schiff base Compound 3.d. Fig. S5. FTIR spectrum of Schiff base Compound 3.e. Fig. S6. FTIR spectrum of Schiff base Compound 3.f. Fig. S7. Normalized UV-vis absorption spectrum of Schiff base 3.a. Fig. S8. Normalized UV-vis absorption spectrum of Schiff base 3.b. Fig. S9. Normalized UV-vis absorption spectrum of Schiff base 3.c. Fig. S10. Normalized UV-vis absorption spectrum of Schiff base 3.d. Fig. S11. Normalized UV-vis absorption spectrum of Schiff base 3.e. Fig. S12. Normalized UV-vis absorption spectrum of Schiff base 3.f. Fig. S13. 1H NMR spectrum of Schiff base Compound 3.a. Fig. S14. 13C NMR spectrum of Schiff base Compound 3.a. Fig. S15. 1H NMR spectrum of Schiff base Compound 3.b. Fig. S16. 13C NMR spectrum of Schiff base Compound 3.b. Fig. S17. 1H NMR spectrum of Schiff base Compound 3.c. Fig. S18. 13C NMR spectrum of Schiff base Compound 3.c. Fig. S19. 1H NMR spectrum of Schiff base Compound 3.d. Fig. S20. 13C NMR spectrum of Schiff base Compound 3.d. Fig. S21. 1H NMR spectrum of Schiff base Compound 3.e. Fig. S22. 13C NMR spectrum of Schiff base compound 3.e. Fig. S23. 1H NMR spectrum of Schiff base Compound 3.f. Fig. S24. 13C NMR spectrum of Schiff base Compound 3.f. [file 13065_2019_642_MOESM1_ESM.docx]

**Benzimidazole Schiff base derivatives: synthesis, characterization and antimicrobial activity**

Thierry Youmbi Fonkui^1^, Monisola Itohan Ikhile^2^, Patrick Berka Njobeh^1^, Derek Tantoh Ndinteh^2^

*^1^Department of Biotechnology and Food Technology, University of Johannesburg* P.O. Box 17011*,* Doornfontein Campus 2028, South Africa*.*

*^2^Department of Applied Chemistry, University of Johannesburg* P.O. Box 17011*,* Doornfontein Campus 2028, South Africa.

**ADDITIONAL DATA**

**Fig. S1 FTIR spectrum of Schiff base Compound 3.a**

**Fig. S2 FTIR spectrum of Schiff base Compound 3.b**

**Fig. S3 FTIR spectrum of Schiff base Compound 3.c**

**Fig. S4 FTIR spectrum of Schiff base Compound 3.d**

**Fig. S5 FTIR spectrum of Schiff base compound 3.e**

**Fig. S6 FTIR spectrum of Schiff base Compound 3.f**

**Fig. S7 Normalized UV-vis absorption spectrum of Schiff base 3.a**

**Fig. S8 Normalized UV-vis absorption spectrum of Schiff base 3.b**

**Fig. S9 Normalized UV-vis absorption spectrum of Schiff base 3.c**

**Fig. S10 Normalized UV-vis absorption spectrum of Schiff base 3.d**

**Fig. S11 Normalized UV-vis absorption spectrum of Schiff base 3.e**

**Fig. S12 Normalized UV-vis absorption spectrum of Schiff base 3.f**


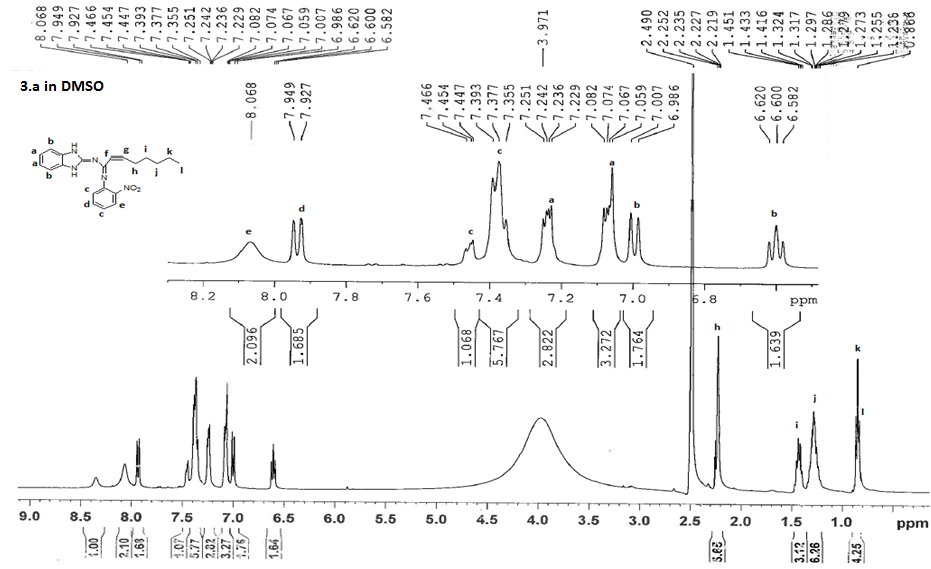


**Fig. S13 ^1^H NMR spectrum of Schiff base Compound 3.a**

**
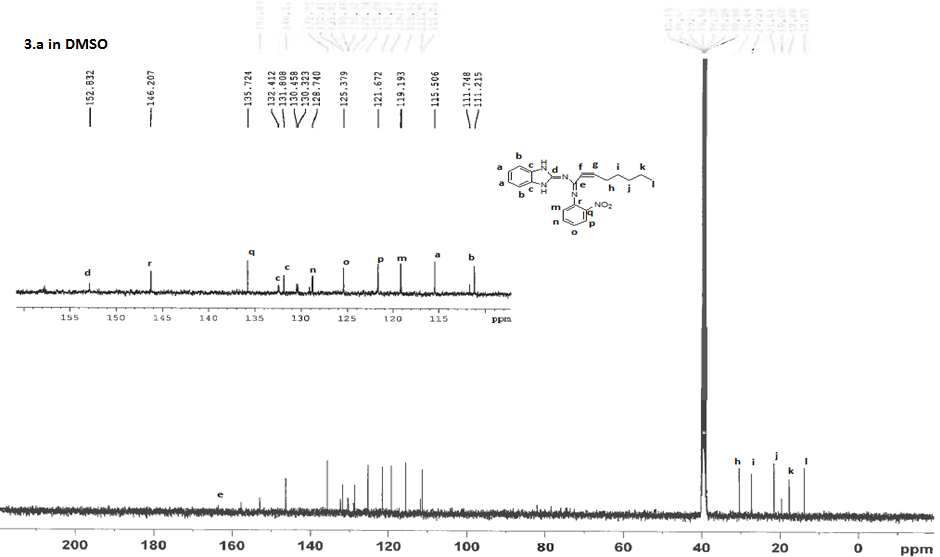
Fig. S14 ^13^C NMR spectrum of Schiff base Compound 3.a**

**
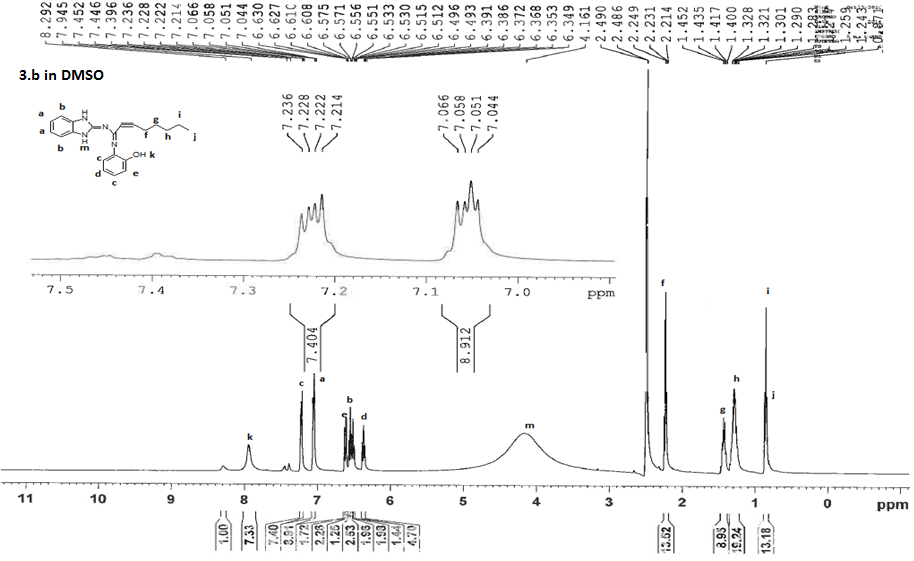
**

**Fig. S15 ^1^H NMR spectrum of Schiff base Compound 3.b**

**
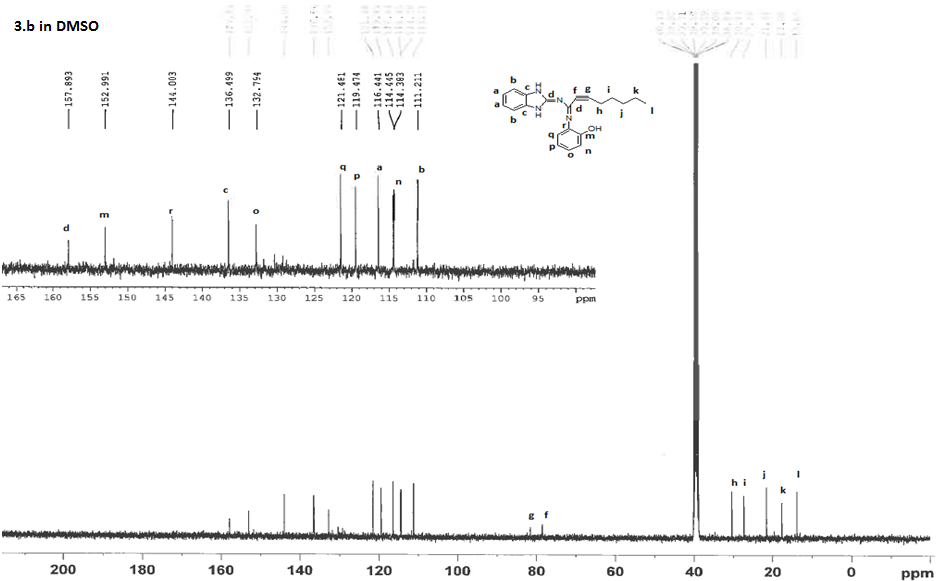
Fig. S16 ^13^C NMR spectrum of Schiff base Compound 3.b**

**
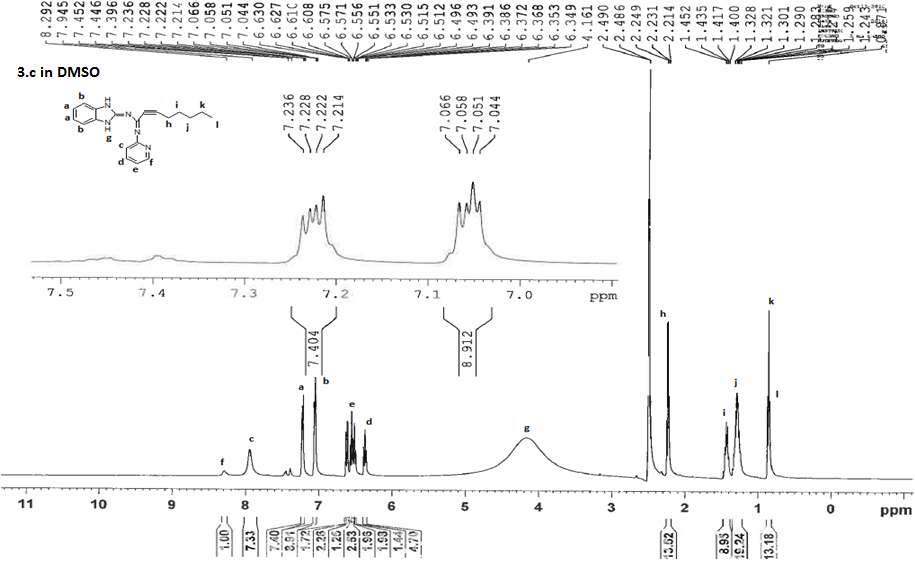
Fig. S17 ^1^H NMR spectrum of Schiff base Compound 3.c**

**
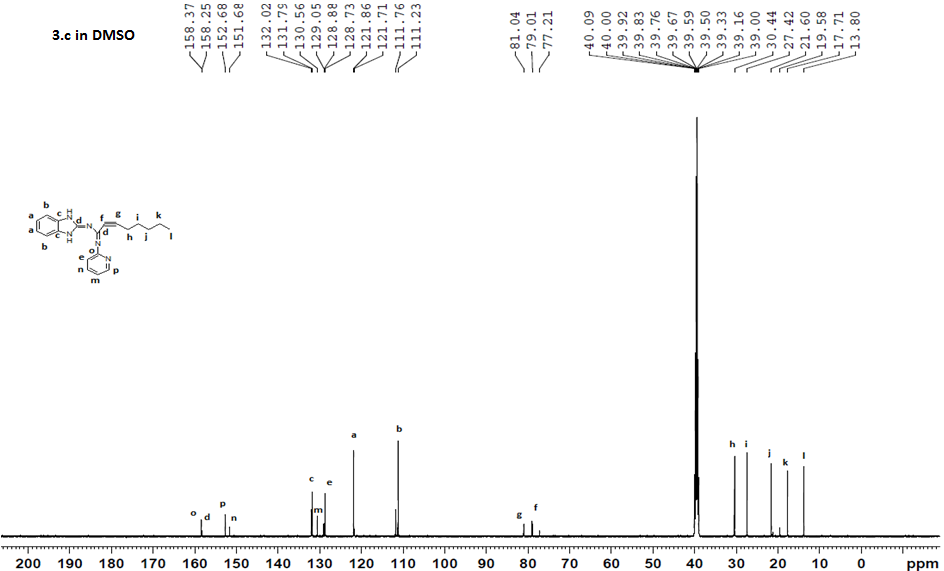
Fig. S18 ^13^C NMR spectrum of Schiff base Compound 3.c**

**
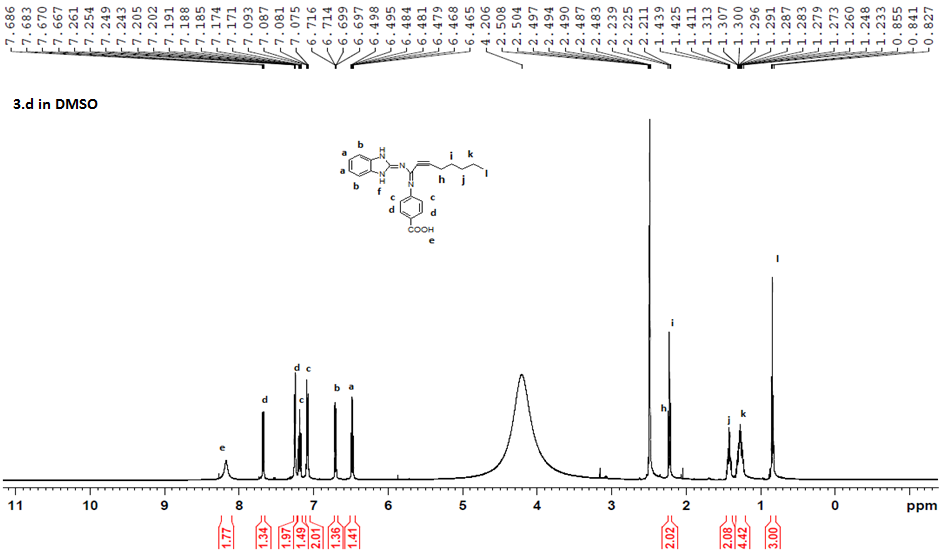
Fig. S19 ^1^H NMR spectrum of Schiff base Compound 3.d**

**
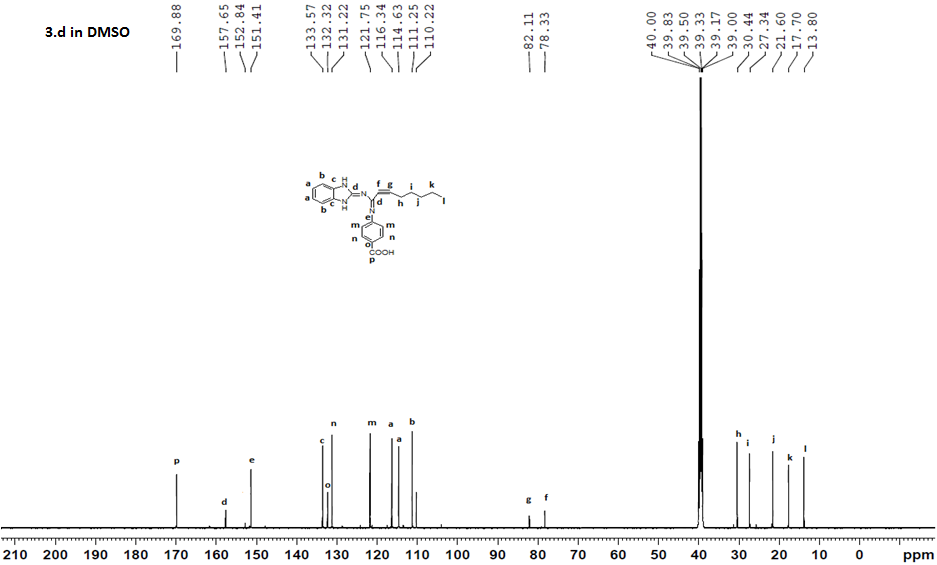
Fig. S20 ^13^C NMR spectrum of Schiff base Compound 3.d**

**
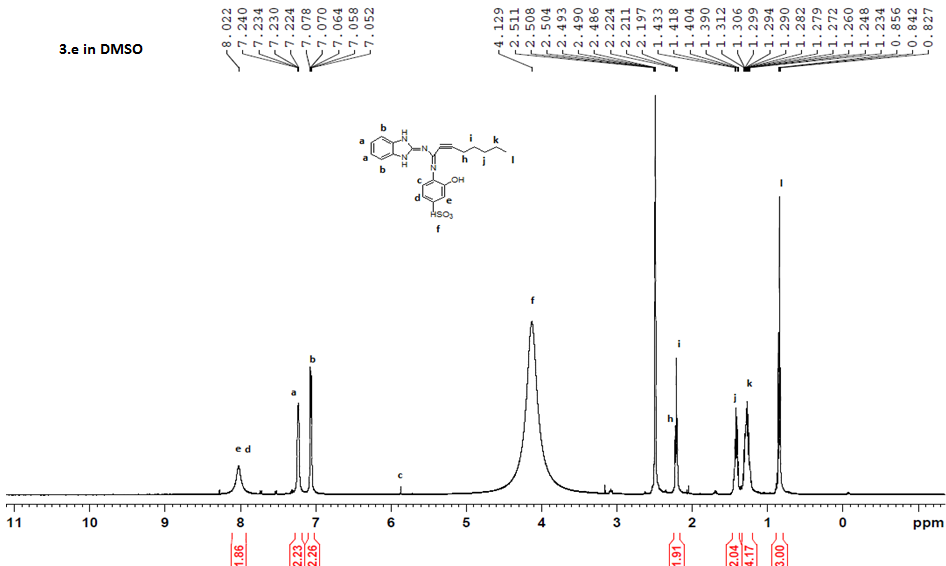
Fig. S21 ^1^H NMR spectrum of Schiff base Compound 3.e**

**
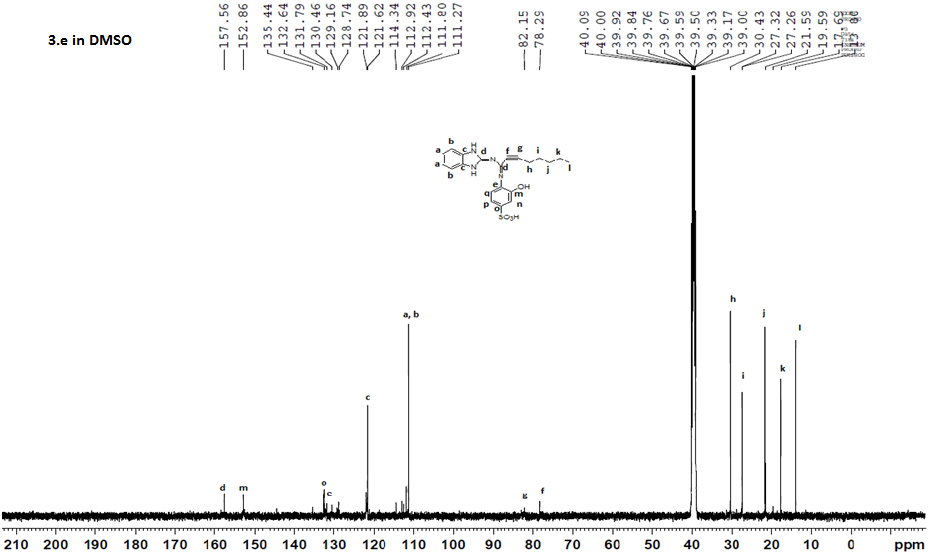
Fig. S22 ^13^C NMR spectrum of Schiff base compound 3.e**

**
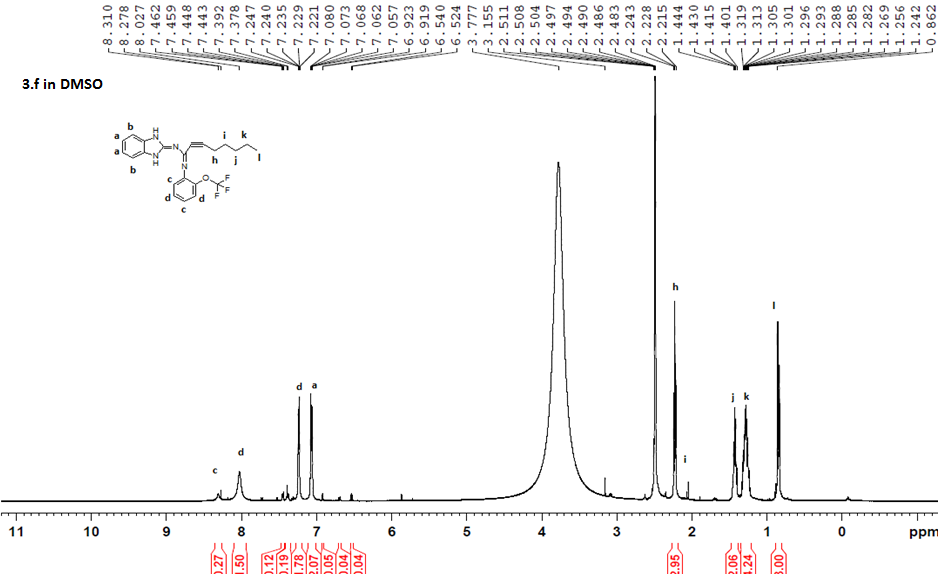
Fig. S23 ^1^H NMR spectrum of Schiff base Compound 3.f**


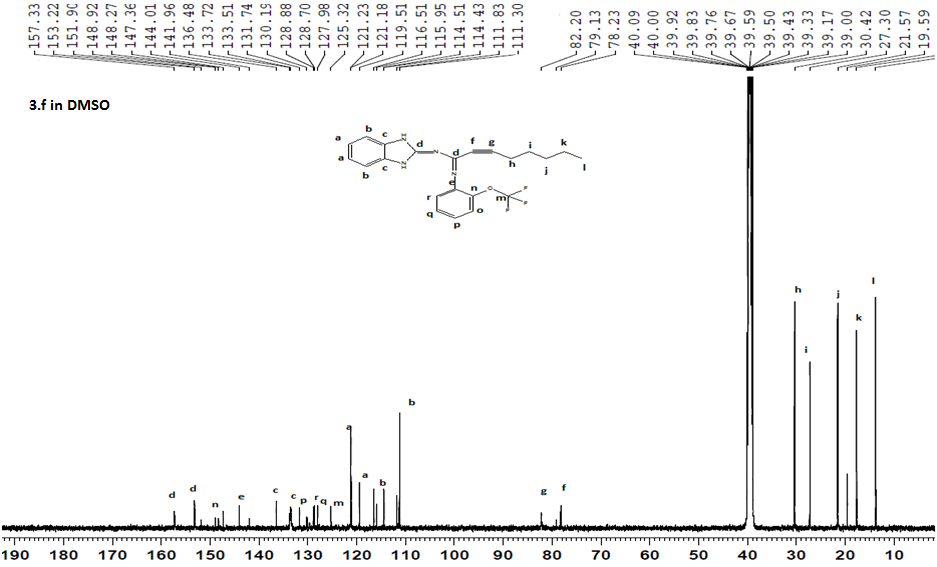
**Fig. S24 ^13^C NMR spectrum of Schiff base Compound 3.f**
